# Correlation Between PD-L2 Expression and Clinical Outcome in Solid Cancer Patients: A Meta-Analysis

**DOI:** 10.3389/fonc.2019.00047

**Published:** 2019-02-13

**Authors:** Huayu Yang, Xiaoxiang Zhou, Lejia Sun, Yilei Mao

**Affiliations:** Department of Liver Surgery, Peking Union Medical College Hospital, PUMC and Chinese Academy of Medical Sciences, Beijing, China

**Keywords:** PD-L2, prognostic biomarker, solid tumor, immune checkpoint, meta-analysis

## Abstract

**Background:** Immune checkpoint inhibitors targeting the programmed cell death 1 (PD-1)/programmed cell death ligand 1 (PD-L1) pathway are a paradigm-shifting cancer therapy. Programmed cell death ligand 2 (PD-L2) is another ligand of PD-1, but its prognostic significance in solid cancer patients after surgery remains controversial. In this study, we aimed to reveal the prognostic implication of PD-L2 in solid tumors through a meta-analysis.

**Methods:** We searched PubMed, Embase and the Cochrane library for studies reporting the relationship between PD-L2 expression and prognosis or clinicopathological features in solid cancer patients after surgery from inception to January 2018, with language restricted to English. Pooled hazard ratios (HRs) and 95% confidence intervals (CIs) were determined to explore the prognostic value of PD-L2 expression. Odds ratios (ORs) were also calculated to investigate the relationship between PD-L2 expression and clinicopathological parameters.

**Results:** Sixteen studies incorporating 3,533 patients were included in our meta-analysis. The pooled results revealed that PD-L2 overexpression was a weak negative predictor for overall survival (OS; HR = 1.38, 95% CI = 1.05–1.81, *P* = 0.021), as well as a strong predictor for poor disease-free survival (DFS)/progression-free survival (PFS) (HR = 1.44, 95% CI = 1.15–1.81, *P* = 0.001). In subgroup analyses, high PD-L2 expression revealed an unfavorable prognostic prediction for OS in hepatocellular carcinoma (HCC) (HR = 1.60, 95% CI = 1.12–2.29, *P* = 0.011) and for DFS/PFS in HCC (HR = 1.50, 95%CI = 1.04–2.16, *P* = 0.031) as well as clear cell renal cell carcinoma (HR = 1.45, 95% CI = 1.03–2.03, *P* = 0.033). Moreover, PD-L2 expression implied a weak trend toward the presence of lymphatic metastasis (presence vs. absence, OR = 1.61, 95% CI = 0.98–2.65, *P* = 0.061).

**Conclusion:** High PD-L2 expression may promote tumor metastasis and predict unfavorable prognosis in solid cancer patients after surgery, especially in HCC.

## Introduction

Over the past decade, one of the most remarkable breakthroughs in cancer therapy has been tumor immunotherapy, in particular immune checkpoint inhibitory programmed cell death 1 (PD-1)/programmed cell death ligand 1 (PD-L1) blockade (1). Anti-PD-1/PD-L1 immunotherapy strengthens antitumor immunity by “releasing the brakes” for immune suppression in the tumor microenvironment and has exhibited inspiring efficacy in various cancer types, resulting in FDA approval and wide clinical application (2–4). PD-1 has been reported to possess two ligands, namely PD-L1 and PD-L2 (5), and the role of the PD-1/PD-L1 axis in cancer has been investigated in depth. It has been reported that PD-L1 predicts unfavorable prognosis in patients with various tumors (6). Nevertheless, PD-L2 has gained little attention, and its function in tumor immunity remains unclear.

Akin to PD-L1, PD-L2 interacts with PD-1 and suppresses T cell proliferation and cytokine release (5). Moreover, PD-L2 can bind to another exclusive partner, namely repulsive guidance molecule b, to promote immune tolerance, although the role of this pathway in cancer is obscure. Contrary to the extensive expression profile of PD-L1, PD-L2 is more confined to antigen-presenting cells, although it can be induced by microenvironmental stimuli (7). Additionally, PD-L2 expression has been discovered in a large number of tumor types (8, 9). One study that analyzed archival tumor samples across seven cancer types showed that in triple-negative breast cancer samples, PD-L2 expression was strongly consistent with PD-L1 expression, with no significantly discordant expression between PD-L1 and PD-L2 in any sample. Among other tumor types, although researchers observed discordant expression in a few samples, PD-L2 expression was also significantly associated with PD-L1 expression (10). While in esophageal adenocarcinoma, PD-L2 expression was identified in approximately half of tumor samples, together with only 2% PD-L1 positivity observed (11). Therefore, although PD-L1 may plays a more dominant role in immunologic modulation of the tumor microenvironment, PD-L2 may have been neglected as a potential target in tumor immunity.

Recently, studies have increasingly focused on the prognostic implications of PD-L2 in cancer patients after surgery. However, whether PD-L2 expression correlates with prognosis in solid cancer patients after surgery remains elusive. Some evidence supports that high PD-L2 expression in cancer specimens predicts impaired survival for various solid tumors (12–16). Nevertheless, several studies report negative results or even opposing findings (11, 17–24). Therefore, we assessed the consistency and magnitude of the prognostic effect of PD-L2 in solid cancer patients after surgery through a meta-analysis. We found that PD-L2 was a negative predictor for prognosis among solid cancer patients. To our knowledge, this study is the first to clarify the prognostic significance of PD-L2 expression in solid tumors by meta-analysis.

## Materials and Methods

### Literature Search Strategy

The implementation of this meta-analysis was conducted in accordance with PRISMA guidelines (25). We systematically reviewed PubMed, Embase, and the Cochrane library for literature published up to January 2018, with the language restricted to English. Keywords adopted to execute the retrieval are as follows: (programmed cell death 1 ligand 2 OR PD-L2 OR B7-DC) AND (cancer OR neoplasm OR malignancy OR carcinoma OR tumor) AND (prognostic OR prognosis). We also checked the references and citations of the retrieved papers. To ensure the reliability of the search results, three authors (HYY & XXZ & LJS) independently executed the retrieval according to the standardized process. Any disagreement between the three authors was resolved through discussion.

### Inclusion and Exclusion Criteria

We adopted the following inclusion criterion: (1) studies published as original articles; (2) studies published in English; (3) studies reporting a correlation between PD-L2 expression in tumor specimens and solid tumor prognosis, such as overall survival (OS), disease-free survival (DFS), and progression-free survival (PFS) or clinicopathological characteristics; (4) available and clear prognostic data from which to directly extract hazard ratios (HR) and 95% confidence intervals (CI); (5) studies had a sample capacity of more than 50 individuals; (6) all enrolled patients received surgery with or without any adjuvant therapy. Studies were excluded if they failed to conform to any inclusion criteria. The analysis of PD-L2 expression in tumor infiltrating lymphocytes was also excluded. For duplicate publications, we included the most informative publication.

### Data Collection and Quality Assessment

Three authors (HYY & XXZ & LJS) independently examined all selected publications and extracted the data in accordance with a standardized protocol. From every study, we pulled the following data: clinicopathological characteristics; first author's name; publication year; country of the patients; number of individuals; cancer type; trial design; cutoff value of high PD-L2; median follow-up time and the range of follow-up time; outcomes of patients; method adopted to probe PD-L2 expression; univariate or multivariate model. If there existed a multivariate HR and a univariate HR, the former one was chosen to avoid the influence of confounding factors.

Also, three authors (HYY & XXZ & LJS) independently assessed the quality of the selected publications on the basis of the Newcastle-Ottawa scale (NOS) criteria (25). Each study was graded on a scale of zero to nine according to the selection, comparability, and outcomes of the study cohorts.

### Statistical Analyses

As DFS and PFS are very similar, we considered the two parameters together. We applied Stata 12.0 software (Stata Corporation, College Station, Texas, USA) to perform the statistical analysis. We applied the log HRs and 95% CIs for aggregation of the prognostic effects (26). We utilized the χ^2^ test and I^2^ statistic to assess the heterogeneity across studies (27). If *P* <0.10 for the χ^2^ test or I^2^ > 50%, significant heterogeneity was considered to exist and the random effects model was utilized (28); If not, a fixed-effects model was utilized (29). We also performed a sensitivity analysis in which one study was deleted every time to judge its impact on the results. We used Begg's funnel plot, Begg's test and Egger's tests to investigate the publication bias quantitatively (30, 31). We utilized the non-parametric “trim and fill” approach to evaluate the potential impact of publication bias, which considered hypothetical negative unpublished studies and recalculated a pooled estimate that comprised these hypothetical studies (32). For all analyses, two-sided *P*-values of <0.05 was regarded as statistically significant.

## Results

### Study Selection

**Figure 1:**
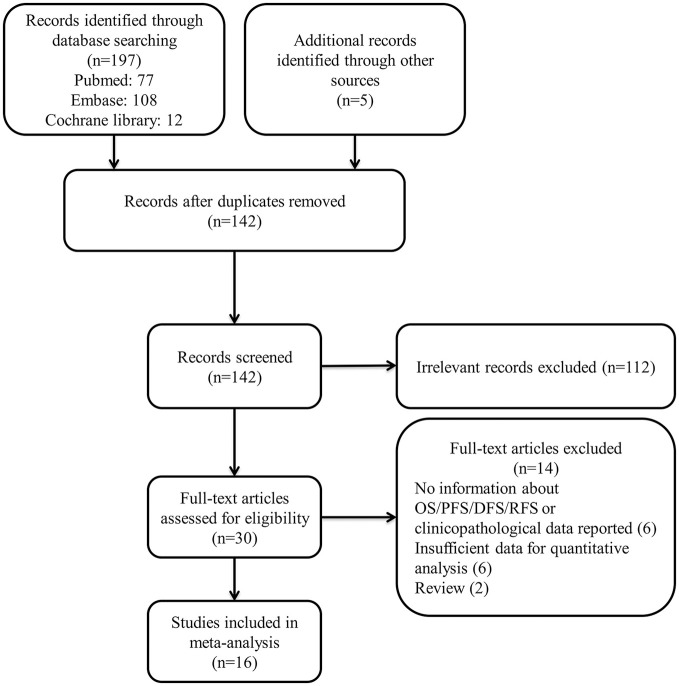
Flow chart of the study screening process.

We selected 142 studies by systematic literature retrieval. After screening the title and abstract, 112 studies were excluded for their irrelevance to the topic. Finally, we included 16 studies after reviewing the full texts (11–24, 33, 34). Studies were excluded for the following reasons: six had no information about OS/DFS/PFS or reported no clinicopathological data; six had insufficient data for quantitative analysis; and two were reviews. The details of our search process are presented in Figure 1.

### Characteristics of the Included Studies

We included data from 3,533 patients in this study. The main characteristics of the 16 studies are summed up in Table 1. One study was published in 2009, and the others were published between 2014 and 2017. Four studies were prospective cohort trials, and 12 were retrospective cohort trials. Patients in all studies were reported to receive resections. These studies were conducted in six countries. With regard to cancer type, studies investigating renal cell carcinoma (RCC; *n* = 3) and gastric cancer (*n* = 3) composed the two largest proportion among all included investigations, followed by hepatocellular carcinoma (HCC; *n* = 2), non-small cell lung cancer (NSCLC; *n* = 2), esophageal cancer (*n* = 2), breast cancer (*n* = 1), oral squamous cell cancer (OSCC; *n* = 1), neurological cancer (*n* = 1), and colorectal cancer (CRC; *n* = 1). More specifically, Two of RCCs were clear cell RCC (ccRCC) and one of RCCs was chromophobe RCC (chRCC); two of gastric cancers were gastric adenocarcinoma (GA) and one of gastric cancers was mixed with tubular adenocarcinoma (TA) and signet ring cell (SRC); one of NSCLCs was squamous cell carcinoma (SqCC) and another one was adenocarcinoma; one of esophageal cancers was SqCC and another was adenocarcinoma. Retrospective trials were designed in 12 studies, and prospective trials were designed in four studies. The cutoff values of high PD-L2 were discordant, while the most common criterion was the median score. With respect to spatial location, all the included studies focus PD-L2 expression on tumor cell. The median follow-up time ranged from 16 months to 7.18 years. The correlation between OS and PD-L2 expression was reported in 13 of the studies. The prognostic value of PD-L2 expression for DFS/PFS was reported in seven studies. In addition, the relationship between clinicopathological features and PD-L2 expression was presented in 13 studies. Seven studies calculated the HRs adjusted for PD-1 expression or PD-L1 expression, and nine studies didn't adjust for PD-1 expression or PD-L2 expression. All included studies used immunohistochemistry (IHC) to examine PD-L2 expression. The origins of PD-L2 antibodies utilized for IHC in the included studies varied, while 7 of the studies used the same antibody (clone 176611, R&D Systems). Nonetheless, only five studies have definitely checked the specificity of the PD-L2 antibody utilized for IHC on a positive control and none of the included studies has set negative control for PD-L2 antibody (Table S1). All included studies were allocated scores >5 on the Newcastle-Ottawa scale (NOS), suggesting that all possessed high methodological quality (Table 2).

**Table 1:** Main characteristics of the eligible studies.

**References**	**Country**	**No. of patient**	**Trial design**	**Cancer type**	**Cancer histology**	**Cutoff value of positive PD-L2**	**Spatial Location**	**Follow-up (months) Medium (range)**	**Outcomes**	**Model (whether adjusted for PD-1 /PD-L1)**	**Method**
Gao (23)	China	240	RC	HCC	HCC	>median values	Tumor, cell	16.0 (1.5–68.0)	OS, DFS	Adjusted	IHC
Zhang et al. (34)	China	143	PC	NSCLC	Adenocarcinoma	>median values	Tumor, cell	N.A.	N.A.	Unadjusted	IHC
Baptista et al. (20)	Brazil	192	RC	Breast cancer	N.A.	>median values	Tumor, cell	86.2	OS, DFS	Adjusted	IHC
Derks et al. (11)	USA	354	RC	Esophageal cancer	Adenocarcinoma	>10% cells positive	Tumor, cell	N.A.	OS	Unadjusted	IHC
Dong et al. (21)	China	855	PC	Gastric cancer	Adenocarcinoma	>median values	Tumor, cell	37.2 (0–93.8)	OS	Unadjusted	IHC
Kim et al. (33)	Korea	331	RC	NSCLC	SqCC	>median values	Tumor, cell	N.A.	N.A.	Unadjusted	IHC
Shin et al. (19)	Korea	91	RC	RCC	ccRCC	scores 2~3	Tumor, cell	34.6 (2.3–171.7)	OS, PFS	Unadjusted	IHC
Shin et al. (19)	Korea	214	RC	RCC	ccRCC	scores 2~3	Tumor, cell	58.7 (1.4–202.1)	PFS	Adjusted	IHC
Tanaka et al. (18)	Japan	180	RC	Esophageal cancer	SqCC	scores 4~9	Tumor, cell	24 (1–196)	OS	Adjusted	IHC
Erlmeier et al., (22)	Germany	81	RC	RCC	chRCC	>median values	Tumor, cell	40.5 (1–226)	OS	Unadjusted	IHC
Gao et al. (13)	China	119	RC	Gastric cancer	Adenocarcinoma	>median values	Tumor, cell	28.0 (4.5–92.0)	OS	Adjusted	IHC
Jung et al. (12)	Korea	85	RC	HCC	HCC	scores 3~5	Tumor, cell	N.A.	OS, DFS	Adjusted	IHC
Kogashiwa et al. (24)	Japan	84	RC	OSCC	SqCC	>5% cells positive	Tumor, cell	40.6 (3.8–89.6)	OS, DFS	Unadjusted	IHC
Pinato et al. (14)	UK	100	PC	Neurological cancer	PCC and PGL	≥5% cells positive	Tumor, cell	56.2 (6–408)	OS	Unadjusted	IHC
Wang et al. (15)	China	124	RC	CRC	N.A.	scores 2~3	Tumor, cell	N.A.	OS, DFS	Unadjusted	IHC
Wu et al. (17)	China	340	PC	Gastric cancer	TA and SRC	scores 3~6	Tumor, cell	48 (1–111)	OS	Adjusted	IHC

*USA, United States of America; UK, United Kingdom; PC, prospective cohort; RC, retrospective cohort; HCC, hepatocellular carcinoma; NSCLC, non-small cell lung cancer; RCC, renal cell carcinoma; OSCC, oral squamous cell carcinoma; CRC, colorectal cancer; SqCC, squamous cell carcinoma; ccRCC, clear cell RCC; chRCC, Chromophobe RCC; PCC, phaeochromocytoma; PGL, paraganglioma; TA, tubular adenocarcinoma; SRC, signet ring cell; IHC, immunohistochemistry; PFS, progression-free survival; DFS, disease-free survival; OS, overall survival; N.A. not available. IHC, immunohistochemistry; Multi, multivariate; Uni, univariate*.

**Table 2:** The Newcastle-Ottawa scale (NOS) quality assessment of the enrolled studies.

**References**	**Selection**				**Comparability**			**Outcome**	**Total**
	**Representativeness of the exposed cohort**	**Selection of the non-exposed cohort**	**Ascertainment of exposure**	**Demonstration that outcome of interest was not present at start of study**	Comparability of cohorts on the basis of the design or analysis (study adjusts for PD-1*, PD-L1*)	**Assessment of outcome**	**Was follow-up long enough for outcomes to occur**	**Adequacy of follow up of cohorts**	
Gao et al. (23)	–	*	*	–	*	*	*	*	6
Zhang et al. (34)	–	*	*	*	–	*	*	*	6
Baptista et al. (20)	–	*	*	–	*	*	*	*	6
Derks et al. (11)	–	*	*	–	–	*	*	*	5
Dong et al. (21)	–	*	*	*	–	*	*	*	6
Kim et al. (33)	–	*	*	–	–	*	*	*	5
Shin et al. (19)^a^	–	*	*	–	–	*	*	*	5
Shin et al. (19)^b^	–	*	*	–	*	*	*	*	6
Tanaka et al. (18)	–	*	*	–	*	*	*	*	6
Erlmeier et al. (22)	–	*	*	–	–	*	*	*	5
Gao et al. (13)	–	*	*	–	**	*	*	*	7
Jung et al. (12)	–	*	*	–	*	*	*	*	6
Kogashiwa et al. (24)	–	*	*	–	–	*	*	*	5
Pinato et al. (14)	–	*	*	*	–	*	*	*	6
Wang et al. (15)	–	*	*	–	–	*	*	*	5
Wu et al. (17)	–	*	*	*	**	*	*	*	8

-,zero score;

* one score;

** two scores;

a *Study by Shin et al. that enrolled 193 ccRCC patients*.

b *Study by Shin et al. that enrolled 425 ccRCC patients*.

### Prognostic Value of PD-L2 Expression for OS

**Figure 2:**
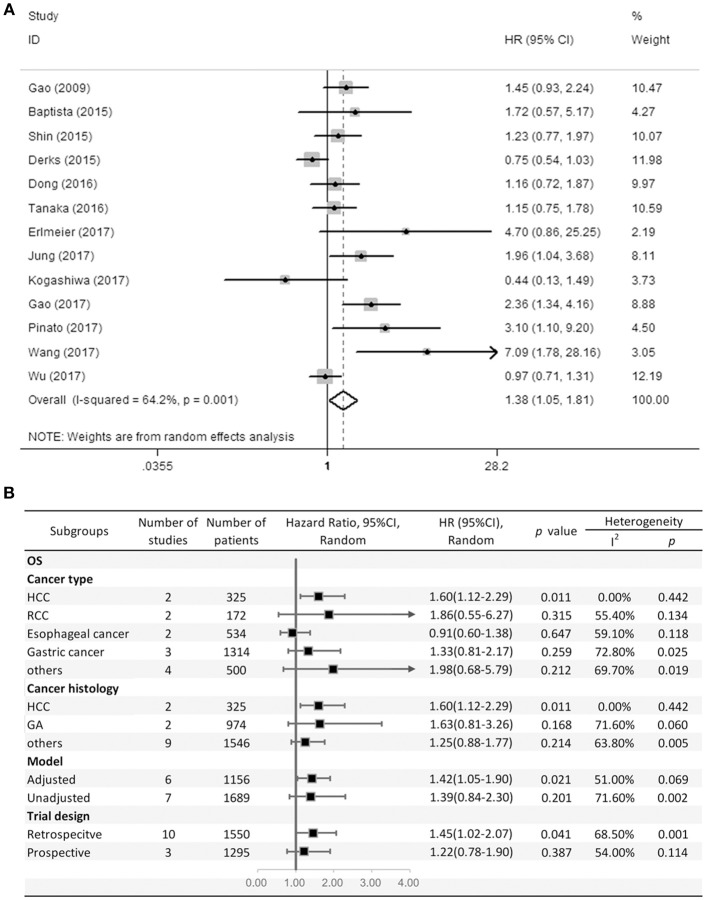
**(A)** Meta-analysis of the association between PD-L2 expression and OS among solid cancer patients after surgery; **(B)** Subgroup analyses of the correlation between PD-L2 and OS.

Thirteen studies consisting of 2,845 patients reported OS. This study revealed that PD-L2 overexpression was a weak negative predictor for OS among patients with various solid tumors (HR = 1.38, 95% CI = 1.05–1.81, *P* = 0.021) (Figure 2A). This pooled meta-analysis was carried out using the random effects model on account of significant heterogeneity (*I*^2^ = 64.2%, *P* = 0.001). To further explore the potential sources of heterogeneity, we utilized subgroup analyses, which are summarized in Figure 2B.

Subgroup analyses regarding cancer type clarified that high PD-L2 expression had a unfavorable prognostic value for OS in patients with HCC (HR = 1.60, 95% CI = 1.12–2.29, *P* = 0.011), while no significant association was observed in RCC, esophageal cancer or gastric cancer (OS for RCC: HR = 1.86, 95% CI = 0.55–6.27, *P* = 0.315; esophageal cancer: HR = 0.91, 95% CI = 0.60–1.38, *P* = 0.647; gastric cancer: HR = 1.33, 95% CI = 0.81–2.17). Notably, since there was only one study each of breast cancer, OSCC, neurological cancer, and CRC, we did not perform subgroup analyses in every cancer type. To further restricted the prognostic effect in homogeneous cancer histology, we performed subgroup analyses according to cancer histology. The pooled results showed that PD-L2 overexpression is not significantly associated with OS in gastric adenocarcinoma (HR = 1.63, 95% CI = 0.81–3.26, *P* = 0.168). By the same token, we didn't perform subgroup analyses in every cancer histology. Moreover, subgroup analyses regarding model and trial design was performed. The pooled results revealed that PD-L2 was a negative predictor for OS among studies adjusted for PD-1 expression or PD-L1 expression (HR = 1.42, 95% CI = 1.05–1.90, *P* = 0.021), whereas it was not significantly correlated with OS among studies not adjusted for PD-1/PD-L1 (HR = 1.39, 95% CI = 0.84–2.30, *P* = 0.201). Similarly, the pooled results were also not consistent between the subgroups divided by trial design (retrospective trial design: HR = 1.45, 95% CI = 1.02–2.07, *P* = 0.041; prospective trial design: HR = 1.22, 95% CI = 0.78–1.90, *P* = 0.387).

### Prognostic Value of PD-L2 Expression for DFS/PFS

**Figure 3:**
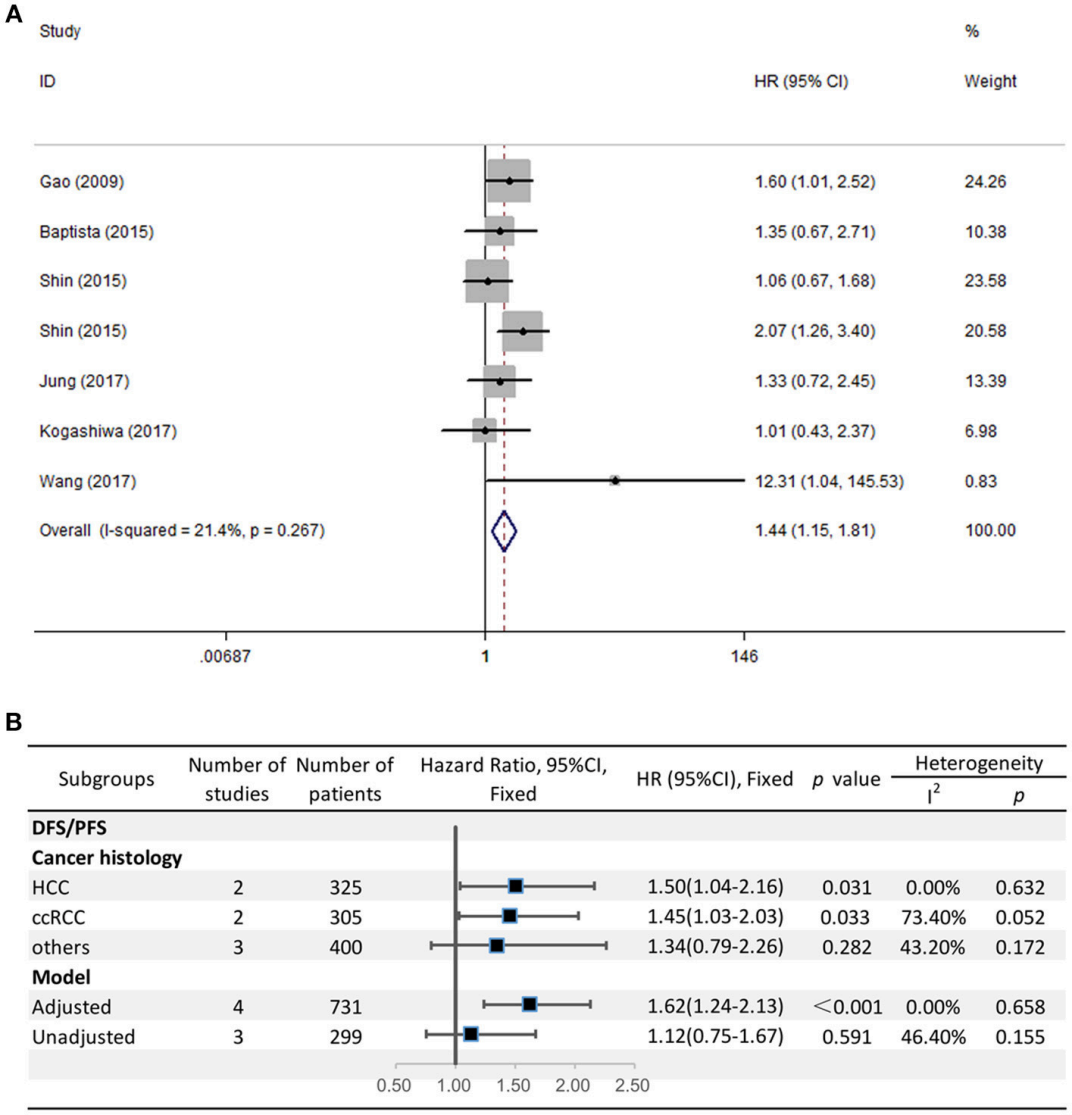
**(A)** Meta-analysis of the association between PD-L2 expression and DFS/PFS among solid cancer patients after surgery; **(B)** Subgroup analyses of the correlation between PD-L2 and DFS/PFS.

The HRs in DFS/PFS were available in seven studies comprising 1,030 patients. Similar to OS, PD-L2 overexpression in tumor samples showed unfavorable DFS/PFS (HR = 1.44, 95% CI = 1.15–1.81, *P* = 0.001) (Figure 3A). The fixed-effects model was applied as no significant heterogeneity was observed (*I*^2^ = 21.4%, *P* = 0.267).

Because the grouping results of cancer type and cancer histology were identical, we directly performed subgroup analyses regarding cancer histology. The stratified analyses indicated that high PD-L2 expression predicted poor DFS/PFS among patients with HCC (HR = 1.50, 95% CI = 1.04–2.16, *P* = 0.031) and ccRCC (HR = 1.45, 95% CI = 1.03–2.03, *P* = 0.033). We did not carry out subgroup analyses for breast cancer, OSCC, or CRC because of the lack of more than one study reporting the associations between prognosis and each cancer histology. The results of subgroup analysis regarding the model for DFS/PFS was consistent with those of OS, namely, PD-L2 was an unfavorable predictor for DFS/PFS among studies adjusted for PD-1/PD-L1 (HR = 1.62, 95% CI = 1.24–2.13, *P* < 0.001), whereas it was not significantly correlated with DFS/PFS among studies not adjusted for PD-1/PD-L1 (HR = 1.12, 95% CI = 0.75-1.67, *P* = 0.591) (Figure 3B).

### Correlation Between PD-L2 Expression and Clinicopathological Characteristics

To comprehensively dissect the role of PD-L2 expression as a biomarker in solid tumors, we explored the correlation between PD-L2 expression and clinicopathological characteristics. Thirteen studies comprising 3,282 patients reported the relationship between PD-L2 expression and clinicopathological parameters. The pooled results indicated that PD-L2 expression implied a weak trend toward the presence of lymphatic metastasis (presence vs. absence, OR = 1.61, 95% CI = 0.98–2.65, *P* = 0.061). Meanwhile, PD-L2 overexpression had no significant association with sex (male vs. female, OR = 1.08, 95% CI = 0.88–1.34, *P* = 0.467), depth of invasion (TIII+TIV vs. TI+TII, OR = 0.99, 95% CI = 0.47–2.01, *P* = 0.467), histopathological stage (III+IV vs. I+II, OR = 0.99, 95% CI = 0.51–1.92, *P* = 0.968), tumor metastasis (presence vs. absence, OR = 1.07, 95% CI = 0.70–1.63, *P* = 0.757), vascular invasion (presence vs. absence, OR = 1.27, 95% CI = 0.67–2.40, *P* = 0.459), recurrence (presence vs. absence, OR = 1.89, 95% CI = 0.72–5.01, *P* = 0.198), differentiation (poor vs. moderate or well, OR = 0.74, 95% CI = 0.48–1.15, *P* = 0.178) or tumor size (≥5 vs. <5 cm, OR = 1.04, 95% CI = 0.72–1.50, *P* = 0.851) (Table 3).

**Table 3:** Meta-analysis of reported clinicopathological characteristics in the included studies.

**Parameters**	**Number of studies**	**Test for association**			**Test for heterogeneity**		
		**OR**	**95% CI**	***p***	***I*^**2**^**	***p***	**Model**
Gender (Male vs. Female)	12	1.08	[0.88–1.34]	0.467	0.0%	0.917	Fixed
Depth of invasion (TIII+TIV vs. TI+TII)	5	0.99	[0.47–2.01]	0.397	79.6%	0.001	Random
Histopathological stage (III+IV vs. I+II)	6	0.99	[0.51–1.92]	0.968	85.2%	< 0.001	Random
Lymphatic metastasis (Presence vs. Absence)	10	1.61	[0.98–2.65]	0.061	78.3%	< 0.001	Random
Tumor metastasis (Presence vs. Absence)	5	1.07	[0.70–1.63]	0.757	0.0%	0.670	Fixed
Vascular invasion (Presence vs. Absence)	5	1.27	[0.67–2.40]	0.459	74.8%	0.003	Random
Recurrence (Presence vs. Absence)	3	1.89	[0.72–5.01]	0.198	69.1%	0.040	Random
Differentiation (poor vs. moderate or well)	6	0.74	[0.48–1.15]	0.178	60.8%	0.026	Random
Tumor size (≥5 cm vs. <5cm)	3	1.04	[0.72–1.50]	0.851	0.0%	0.380	Fixed

*OR, odds ratio*.

### Sensitivity Analysis and Publication Bias

**Figure 4:**
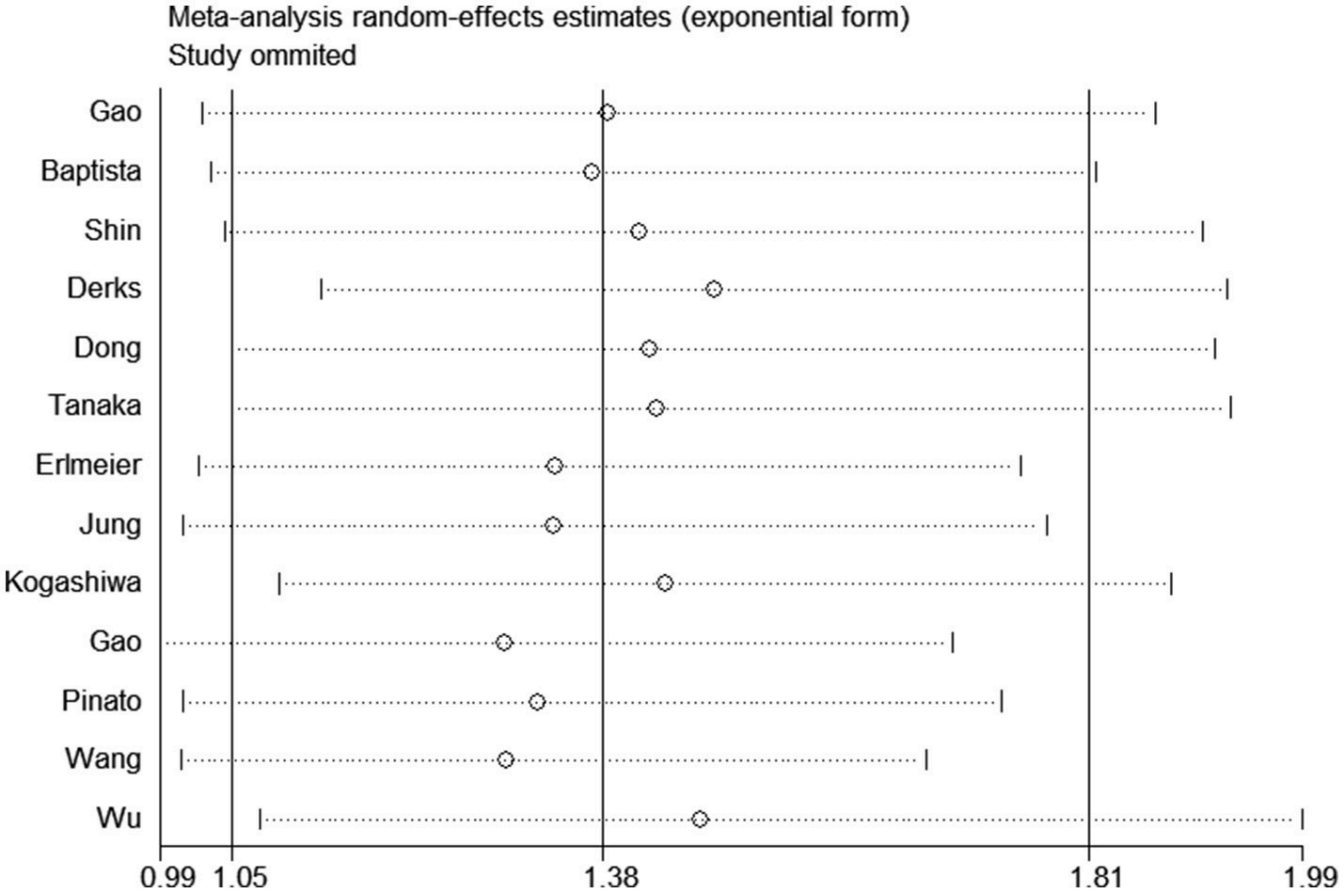
Sensitivity analysis of the effect of individual studies on the pooled HRs for PD-L2 and OS.

**Figure 5:**
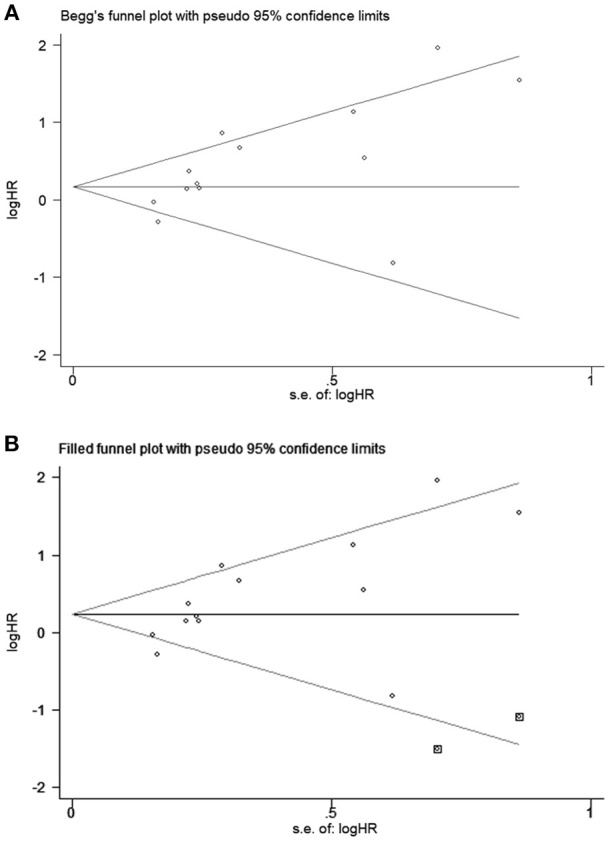
Begg's funnel plots for publication bias of PD-L2 on OS. **(A)** Publication bias for OS without trim and fill; **(B)** publication bias for OS with trim and fill.

**Figure 6:**
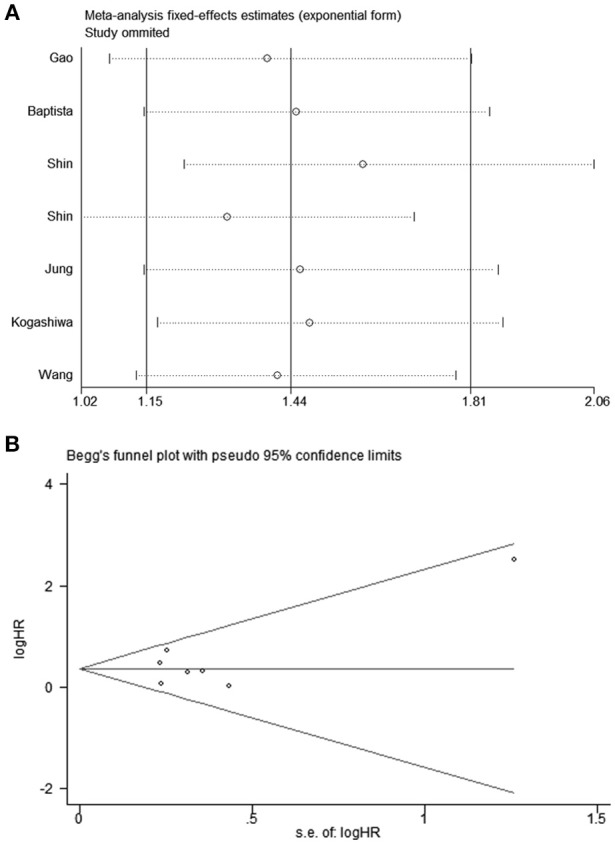
**(A)** Sensitivity analysis of the effect of individual studies on the pooled HRs for PD-L2 and DFS/PFS; **(B)** Funnel plots for publication bias of PD-L2 on DFS/PFS.

All studies were successively omitted to judge the robustness of the pooled results. Furthermore, we combined Begg's funnel plot, Begg's test and Egger's test to evaluate whether a publication bias existed. As shown in Figure 4, the significance of the recalculated HRs did not change when any study was deleted except for Gao's study, which was published in 2017. When Gao's study was omitted, the HRs of the pooled OS became borderline to the statistical threshold (HR = 1.29, 95% CI = 0.99–1.69, *P* = 0.063), which indicated that the correlation between PD-L2 expression and OS was not robustly significant. Of note, Egger's test of OS indicated the existence of publication bias (*P* = 0.019), and visual estimation of Begg's funnel plot of OS revealed evident asymmetry, together with the results that Begg's test of OS was closed to the threshold of statistical significance (*P* = 0.059; Figure 5A). Hence, we performed a further sensitivity analysis with the trim and fill method to evaluate the effect of the potential publication bias of OS. As shown in Figure 5B, two hypothetical negative studies were added to diminish the asymmetry of Begg's funnel plot. However, the pooled results comprising the hypothetical resulting in losing the statistical significance of the correlation between PD-L2 expression and OS (HR = 1.274, 95% CI = 0.96–1.69). Therefore, we must be careful in drawing a conclusion regarding OS. As to DFS/PFS, the significance of the recalculated HRs did not change when any study was deleted (Figure 6A). In addition, the shape of the Begg's funnel plot did not display evident asymmetry (Figure 6B), indicating that no obvious publication bias existed, which was confirmed by Begg's test (*P* = 0.764) and Egger's test (*P* = 0.409).

## Discussion

Recently, a growing number of studies have investigated the prognostic implication of PD-L2 protein expression in tumors among patients with solid tumors, and the results remain controversial. A transcriptomic analysis performed by Danilova and colleagues used The Cancer Genome Atlas datasets and found that PD-L2 mRNA expression indicated good prognosis in skin cutaneous melanoma (SKCM) but had no prognostic role in other 8 tumors (35). However, mRNA expression may not accurately represent the protein expression. In this meta-analysis, we included studies reporting PD-L2 protein expression and revealed that PD-L2 overexpression was a weak negative predictor for OS and a strong negative predictor for DFS/PFS in solid cancer patients after surgery. Similarly, PD-L2 displayed inconsistent prognostic significance in different cancer types and histology. PD-L2 positive expression showed unfavorable prognostic prediction for OS in HCC and for DFS/PFS in HCC and ccRCC; however, the prognostic value remained not significant for other cancer types and histology. In addition, high PD-L2 expression implied a weak trend toward the presence of lymphatic metastasis. Taken together, high PD-L2 expression might promote tumor metastasis and predict unfavorable prognosis in solid cancer patients after surgery, especially in HCC.

Contrary to PD-L1, PD-L2 is less well-studied, and the underlying mechanism involved in the correlation between PD-L2 positive expression and decreased survival remains unclear. Previous studies showed that PD-L2 exerts its main physiological and pathological function in immune tolerance via dampening and modulating T helper type 2 (Th2) response (36–38). However, Th1 immuno-responses are the most dominant in the context of antitumor immunity. In esophageal squamous cell carcinoma (ESCC), PD-L2 was shown to negatively associate with the number of PD-1+ tumor infiltrating lymphocytes (TILs), indicating that PD-L2 expressed on ESCC cells might restrain PD-1+ TILs activity, ultimately facilitating immune escape (39). This inverse correlation between PD-L2 expression and abundance of TILs has also been reported elsewhere (40). Recently, a multivariate analysis revealed that in CRC, high tumor PD-L2 expression was correlated with a weak Crohn's-like lymphoid reaction, which was deemed to play a significant role in adaptive immune responses against tumors, suggesting that PD-L2 expression might reduce Crohn's-like lymphoid reactions to suppress antitumor immunity (41). Moreover, Pinato et al. revealed that PD-L2 upregulation was associated with tumor hypoxia (14). As tumor hypoxia is generally recognized as a contributor to immune resistance in the tumor microenvironment (42), it is conceivable that PD-L2 may potentially mediate immune resistance via promoting tumor hypoxia. Furthermore, high PD-L2 expression in tumor tissue was independently associated with better clinical response to pembrolizumab in patients with head and neck squamous cell carcinoma, suggesting that PD-L2 expression in tumor tissue may exert a vital role in response to PD-1 blockade therapy, although this study was not included in our meta-analysis (10). However, whether PD-L2 has a causal relationship with immune suppression in tumor immunity as well as the underlying mechanism requires further investigation.

In recent years, immune checkpoint inhibitors, particularly those targeting the PD-1 pathway, have become a paradigm-shifting therapy in cancer treatment. Blockade of PD-L2 in a pancreatic murine model also displayed evident anti-tumor effects with decreased tumor outgrowth rates (43). In contrast, a PD-L2 knockout mouse bearing a CT26 tumor exhibited a weakened tumor-specific cytotoxic T lymphocyte response and more rapid tumor growth compared with a WT mouse bearing CT26 (44). These inconsistent outcomes leave the targeting potential of PD-L2 unresolved. Recently, Ahmad and colleagues described CD4+ and CD8+ PD-L2-specific T cells, which can directly exert cytotoxic activity against PD-L2-expressing target cells and indirectly release pro-inflammatory cytokines into the tumor microenvironment, indicating the possibility of novel PD-L2-based vaccines complementary to checkpoint inhibitors. The results of our meta-analysis further suggest that PD-L2 is a pejorative prognostic biomarker for solid tumors, especially in HCC. Further investigations studying the mechanism of the role of PD-L2 in solid tumor immunity are required to understand the targeting potential of PD-L2.

Regarding the tumor types and tumor histology, the unfavorable prognostic effect of PD-L2 was consistently significant both for OS and DFS/PFS in HCC, which has startling heterogeneity and lacks an efficient therapeutic approach (45, 46), suggesting that PD-L2 may serve as prognostic marker especially in HCC. However, in esophageal cancer, high PD-L2 expression implied a favorable prognosis trend, although with no statistical significance. This discordant result suggested that perhaps PD-L2 has different effects on immune suppression among different cancer types. More studies reporting the prognostic roles of PD-L2 in specific cancer types and histology are thus required. Given that PD-L2 expression is widely reported to be correlated with PD-L1 expression and PD-L1 has been reported to predict pejorative prognosis in solid tumors (6, 10), it is crucial to exclude the confounding effect of PD-L1 before we draw conclusion for PD-L2. Indeed, subgroup analyses regarding model revealed that the prognostic role of PD-L2 for both OS and DFS/PFS remained significant in studies adjusted for PD-1 expression or PD-L1 expression, indicating that PD-L2 overexpression predicts poor prognosis in solid tumors independent of PD-1/PD-L1 expression and other confounding factors. Of note, all studies included in our study utilized IHC to determine PD-L2 expression in specimens. IHC is the major approach by which to probe the expression level of protein in resected tumor samples and has been widely applied. Therefore, our findings are very feasible for clinical practice.

Our study had several limitations. First, sensitivity analyses revealed that the correlation between PD-L2 expression and OS was not robustly stable, which might be explained by the publication bias that existed in this meta-analysis. To validate our hypothesis, we utilized the trim and fill method to include two hypothetical negative results, revealing that the correlation between PD-L2 expression and OS lost significance. It seemed that because positive findings tend to be published compared with negative findings, the association between PD-L2 expression and OS might potentially be exaggerated. Nevertheless, the association between PD-L2 expression and DFS/PFS was robust and stable, with no publication bias observed. Considering that PD-L2 expression has an unstable weak significant prognostic effect for OS and stable significant prognostic effect for DPS/PFS, we believe that our study reveals meaningful statistical evidence endorsing the prognostic value of PD-L2 to predict unfavorable outcome in solid cancer patients after surgery. Second, the OS results displayed heterogeneity in our meta-analysis, which may be because of the different analysis strategy of IHC and heterogenous cut-off values of PD-L2 expression. However, we performed subgroup analyses in an attempt to minimize the impact of heterogeneity. We found that PD-L2 was an unfavorable predictor of OS in a homogeneous HCC population. In addition, because of the extensively heterogenous cut-off values of PD-L2 expression, we were unable to stratify the studies according to cut-off values. Furthermore, most of the included studies didn't check the specificity of PD-L2 antibody utilized for IHC, which might weaken the reliability of the result. Thus, to remove the heterogeneity in current clinical protocols, a unified approach for protein expression assessment and a rigorous check for antibody specificity are urgently required. Last, all studies selected for this meta-analysis focused on PD-L2 expressed by tumor cells. However, whether PD-L2 expressed by tumor cells or other cells in the tumor microenvironment plays the dominant role is unclear. PD-L2 has also been found to be expressed by stromal cells and appears to be functional (47, 48). It should be noted that perhaps not only PD-L2 expression in tumor cells but also its expression in stromal cells plays a significant role in immune suppression and affects prognosis. We highly recommend further studies to investigate the prognostic roles of PD-L2 expressed by stroma cells in addition to tumor cells.

In conclusion, our study has clinical significance because it clarifies the correlation between high PD-L2 expression and unfavorable prognosis for solid cancer patients after surgery, especially in HCC.

## Author Contributions

HY designed the study, performed the literature search and screening, performed the data analyses and wrote the manuscript. XZ designed the study, retrieved the literature and data, analyzed the retrieved data and participated in the writing of manuscript. LS assisted in the designing of the study, performed the literature search and screening, assisted in the data analyses and participated in the writing of manuscript. YM designed the study and supervised the study. Every author approved the final version of this study.

### Conflict of Interest Statement

The authors declare that the research was conducted in the absence of any commercial or financial relationships that could be construed as a potential conflict of interest.
