# Supplementary material for: Correlation Between PD-L2 Expression and Clinical Outcome in Solid Cancer Patients: A Meta-Analysis
Source: Front Oncol. 2019 Feb 13;9:47. doi: 10.3389/fonc.2019.00047 (PMC6413700; doi:10.3389/fonc.2019.00047)
Supplement: Supplementary file 1 [file Table_1.pdf]

Table S1: Origins of the PD-L2 antibodies utilized for IHC in the included studies.

**Author**	**Year**	**Clone of PD-L2 antibody**	**Manufacturer of PD-L2 antibody**	**Positive control for PD-L2 antibody**	**Negative control for PD-L2 antibody**
Gao	2009	N.A.	R&D Systems	No	No
Zhang	2014	Polyclonal	Sigma-Aldrich	No	No
Baptista	2015	Polyclonal	Abcam	Liver tissue	Uncertain
Derks	2015	366C.9E5	Dr Gordon Freeman's lab	No	No
Dong	2015	Polyclonal	Abcam	Slides with known positive reactivity	No
Kim	2015	176611	R&D Systems	Placental and tonsil	No
Shin[a]	2015	176611	R&D Systems	No	No
Shin[b]	2015	176611	R&D Systems	No	No
Tanaka	2016	176611	R&D Systems	Placental tissue	No
Erlmeier	2017	176611	R&D Systems	Internal vascular endothelial cells	No
Gao	2017	176611	R&D Systems	No	No
Jung	2017	N.A.	N.A.	No	No
Kogashiwa	2017	80380	Sigma-Aldrich	No	No
Pinato	2017	Polyclonal	Sigma-Aldrich	No	No
Wang	2017	N.A.	Abcam	No	No
Wu	2017	176611	R&D Systems	No	No

N.A. not available; [a]Study by Shin et al. that enrolled 193 RCC patients; [b]Study by Shin et al. that enrolled 425 RCC patients.
